# The glial response to intracerebrally delivered therapies for neurodegenerative disorders: is this a critical issue?

**DOI:** 10.3389/fphar.2014.00139

**Published:** 2014-07-10

**Authors:** Francesca Cicchetti, Roger A. Barker

**Affiliations:** ^1^Axe Neurosciences, Centre de Recherche du CHU de QuébecQuébec, QC, Canada; ^2^Département de Psychiatrie et Neurosciences, Université LavalQuébec, QC, Canada; ^3^John van Geest Centre for Brain Repair, Department of Clinical Neuroscience, University of CambridgeCambridge, UK

**Keywords:** gene therapy for neurodegenerative diseases, cell transplantation, deep brain stimulation, Alzheimer's disease, Huntington's disease, Parkinson's disease, astrocytes, microglia

## Abstract

The role of glial cells in the pathogenesis of many neurodegenerative conditions of the central nervous system (CNS) is now well established (as is discussed in other reviews in this special issue of Frontiers in Neuropharmacology). What is less clear is whether there are changes in these same cells in terms of their behavior and function in response to invasive experimental therapeutic interventions for these diseases. This has, and will continue to become more of an issue as we enter a new era of novel treatments which require the agent to be directly placed/infused into the CNS such as deep brain stimulation (DBS), cell transplants, gene therapies and growth factor infusions. To date, all of these treatments have produced variable outcomes and the reasons for this have been widely debated but the host astrocytic and/or microglial response induced by such invasively delivered agents has not been discussed in any detail. In this review, we have attempted to summarize the limited published data on this, in particular we discuss the small number of human post-mortem studies reported in this field. By so doing, we hope to provide a better description and understanding of the extent and nature of both the astrocytic and microglial response, which in turn could lead to modifications in the way these therapeutic interventions are delivered.

## Introduction

Over the last 30 years, there has been a burgeoning of new therapeutic approaches to treat chronic neurodegenerative conditions of the central nervous system (CNS). These approaches have essentially been of two main types:

- Better symptomatic agents most notably deep brain stimulation (DBS) for Parkinson's Disease (PD) as well as gene therapy approaches (e.g., AAV2-GAD; ProSavin®; hAADC gene therapy).
- Restorative agents such as growth factor administration or cell transplants. This has involved either directly infusing growth factors into the CNS (e.g., GDNF in the case of PD) or the use of viral delivery systems (e.g., AAV2-Neurturin). In the case of cell therapies a number of different cell types have been grafted into the diseased brain especially in patients with PD (e.g., adrenal medulla; carotid body; ventral mesencephalon, amongst others) and Huntington's Disease (HD) (fetal striatal cells).

Whilst the efficacy of these approaches has been the subject of many reviews, one area that has received rather less attention is the host response to these therapeutic agents. This involves not only an anticipated and beneficial response (e.g., cell integration, fiber sprouting, synapse formation, and so on) but also a glial reaction to it, involving both astrocytes and microglia. In the adult CNS, astrocytes constitute the predominant glial cell type that function to control the CNS environment by removing excess ions and recycling neurotransmitters, supporting endothelial cells that form the blood-brain-barrier, as well as playing a key role in tissue scarring and repair following injury. In recent years, the importance of cross-communication between astrocytes, as well as between astrocytes and neuronal cells has started to be better understood. Microglial cells, in contrast, have more of a phagocytic function but like astrocytes, are known to release cytokines in inflammatory/infectious conditions. Whilst both astrocytes and microglia react to, and convey messages within their immediate environment, they also can communicate over extended distances through syncytial networks.

In this review, we will discuss the extent to which the glial response may limit, or possibly augment, the potential effects of intracerebrally delivered therapeutic approaches for neurodegenerative disorders. The focus of this mini review will be largely restricted to the astrocytic and microglial responses in human post-mortem studies, as there is no published data on the oligodendroglial response to these types of treatments. However, because of the importance of pre-clinical data in this field, we have also summarized studies that have reported on the nature of the glial response in the context of DBS (Table 1), neurotrophic therapies (Table 2), and neural grafting (Table 3) *in vivo*.

**Table 1:** **Glial response to DBS and electrode implantation in *in vivo* models**.

**References**	**Species**	**Condition**	**Target site**	**Type of stimulation**	**Period of experimentation/observation**	**Nature of the glial response**
Han et al., 2012	Cat	Normal	Inferior colliculus	Chronic	3–18 months	Observable astrogliosis near the probe at 2 months, but reduced at 5 months
Morimoto et al., 2011	Male Wistar rat	Stroke	Striatum	Chronic	1 week	Diminished microglial activation with stimulation
Hirshler et al., 2010	Male Sprague-Dawley rat	Normal	Subthalamic nucleus	Electrode implantation only	1–8 weeks	Astrocytic and microglial activation; more significant in cortex, striatum, and thalamus
Baba et al., 2009	Wistar rat	Stroke	Cortex (ischemic boundary)	Chronic	1 week	Diminished microglial and astrocytic proliferation
Harnack et al., 2008	Male Wistar rat	Normal	Subthalamic nucleus	Chronic	3 weeks	Increased number of GFAP^+^ astrocytes at all anatomical sites as well as thickening of processes
Leung et al., 2008	Rat	Normal	Cortex	Electrode implantation only	12 weeks	Observable activated microglia attached to the electrodes' external coatings
Biran et al., 2007	Male Fischer-344 rat	Normal	Cortex	Electrode implantation only	2–4 weeks	Microglia and astrocyte activation around the electrode
Lenarz et al., 2007	Cat	Normal	Inferior colliculus	Semi-chronic (4 h/day)	Implantation: 3 months Stimulation: 60 days beginning 4 weeks post-implantation	Increased GFAP^+^ cell density around the electrode (greater with the stimulated than the non-stimulated electrode)
Stice et al., 2007	Female Sprague-Dawley rat	Normal	Cortex	Electrode implantation only	2 and 4 weeks	Astrocytic scar around the electrodes
Griffith and Humphrey, 2006	Rhesus macaque	Normal	Cortex	Electrode implantation only	3 months and 3 years	Persistent reactive astrogliosis around the electrodes (3 months to 3 years). Transient microglial reaction (present at 3 months but not at 3 years)
Biran et al., 2005	Male Fischer-344 rat	Normal	Cortex	Electrode implantation only	4 weeks	Persistent activated microglia around the electrode
Kim et al., 2004	Male Fischer-344 rat	Normal	Striatum	Electrode implantation only	4 weeks	Significant increase in activated microglia in all brain regions

**Table 2:** **Glial response to neurotrophic therapies in *in vivo* models**.

**References**	**Species**	**Condition**	**Vector**	**Delivery site**	**Nature of the glial response**
Rahim et al., 2012	MF1 mouse (fetal)	Normal	Ad5 and AAV pseudotypes 2/5, 2/8, 2/9	Lateral ventricle (trans-uterine injection)	No significant microglia-mediated immune response (with any of the vectors)
Louboutin et al., 2011	Rhesus macaque	Normal	Recombinant SV40-derived vector	Caudate nucleus	No microglia or astrocyte reactions
Rahim et al., 2011	MF1 mouse (fetal and neonatal)	Normal	AAV pseudotype 2/9	Intravenous	No microglia-mediated immune response
Hadaczek et al., 2010	Male Rhesus macaque	PD (MPTP lesion)	AAV	Striatum	No signs of neuroinflammation or reactive gliosis up to 8 years
Lattanzi et al., 2010	Mouse	Globoid cell or metachromatic leukodystrophy	Lentivirus (coding for beta-galactocerebrosidase or arylsulfatase A)	External capsule	Decrease in activated astrocytes and microglia
Snyder-Keller et al., 2010	B6.HDR6/1 mouse	HD	AAV2/1 (delivering anti-htt scFv-C4)	Striatum	Modest glial reaction (activated microglia) at the injection site
Toupet et al., 2008	C57Bl/6J mouse	Prion disease	Lentivirus	Hippocampus	Remarkable decrease in astrogliosis
Louboutin et al., 2007	Female Sprague-Dawley rat	Normal	Recombinant SV40-derived	Caudate-putamen or lateral ventricle	Increased number of astrocytes along the needle track (suggested to be reparative gliosis in response to the minor lesion provoked by the needle)
Zou et al., 2001	Male Fischer-344 rat	Aged brain	hdAdv and fgAdv	Intraventricular or hippocampus	Activation of microglia and astrocytes at injection sites: lower with hdAdv than with fgAdv
Driesse et al., 1998	Rhesus macaque	Normal	Adenovirus	Frontal lobe white matter (unilateral)	Astrocyte activation

*Abbreviations: AAV, Adeno-associated virus; Ad5, Adenovirus serotype 5; fgAdv, first-generation adenoviral vectors; HD, Huntington's disease; hdAdv, Helper-dependent adenovirus; htt, huntingtin; MPTP, 1-methyl-4-phenyl-1,2,3,6-tetrahydropyridine; PD, Parkinson's disease*.

**Table 3:** **Glial response to neural grafting in *in vivo* models**.

**References**	**Species**	**Condition**	**Cell type**	**Implantation site**	**Nature of the glial response**
De Vocht et al., 2013	Male FVB/NCrl mouse	Normal	Autologous mesenchymal stromal cells	Right hemisphere	M1-microglia and severe astrogliosis surrounding the graft (2 weeks post-tp)
Ma et al., 2013	APP/PS1 double transgenic mouse	AD	Adipose-derived mesenchymal stem cells	Hippocampus and cortex	Increased number of activated microglia in transplanted regions
Osman et al., 2013	Mouse	Irradiated	Syngenic enteric neural stem/progenitor cells	Hippocampus	Microgliosis and astrogliosis associated with grafted cell clusters
Tripathy et al., 2013	Male Sprague-Dawley rat	PD	Differentiated neurons from murine embryonic stem cells	Striatum	Increased expression of microglia-derived factors (CD11b and Iba1). Astrocytosis in the grafted region. Increase in GDNF
Mosher et al., 2012	C57Bl/6J mouse	Normal	Mouse neural progenitor cells	Striatum	Increased number of Iba1^+^ microglia in transplanted regions
Praet et al., 2012	C56Bl/6 mouse	Normal and cuprizone-treated	Neural stem cells	Below the capsula externa	Extensive invasion of GFAP^+^ astrocytes and Iba1^+^ microglia (few CD11b^+^) within graft sites. Astrocytic scar surrounding graft
Khoo et al., 2011	Wistar Ob rat	PD (6-OHDA lesion)	Bone marrow-derived human mesenchymal stem cells (undifferentiated and neuronal-primed)	Striatum and substantia nigra	Iba-1^+^ microglia and GFAP^+^ astrocytes surrounding the grafts (7 days post-tp)
Coyne et al., 2006	Female Sprague-Dawley rat	Normal	Allogeneic marrow stromal cells	Hippocampus or striatum	Massive infiltration of ED1^+^ microglia leading to graft rejection. Marked astrogliosis surrounding grafts
Muraoka et al., 2006	Male Fischer 344 rat	Normal	Autologous vs. allogeneic neural stem cells	Hippocampus	Astrocyte and microglia reactivity in the host tissue (lower in autologous than in allogeneic tp)
Jiang et al., 1999	Monkey	PD	Microencapsulated rat myoblasts transfected with the tyrosine hydroxylase gene	Striatum	No obvious gliosis around microcapsules
Dunnett et al., 1998	R6/2 mouse	HD	Syngenic striatal cell suspension	Striatum	Modest astroglial reaction at the graft-host border
Pennell and Streit, 1997	Rat	Normal	Embryonic neural cell suspension (whole, or microglial and endothelial cell-depleted)	Striatum	Ameboid microglial cells within grafts early post-tp. By 30 days post-tp, microglia display a resting phenotype within grafts
Barker et al., 1996	Female Sprague-Dawley rat	PD (6-OHDA lesion)	Embryonic ventral mesencephalic tissue	Striatum	Transient astrogliosis and microglial reaction surrounding grafts
Kosno-Kruszewska et al., 1996	Rat	Normal	Cryopreserved ventral mesencephalic tissue	Striatum	Similar glial scar in both grafted and sham-lesion conditions
Duan et al., 1995	Female Sprague-Dawley rat	Normal	Dissociated embryonic ventral mesencephalic: (murine syngeneic, allogeneic, or xenogeneic)	Striatum	Similar reactions in syngeneic and allogeneic: activated microglia infiltration on day 4, decreasing at 6 weeks. More intense reaction in xenografts leading to rejection
Helm et al., 1993	Female Rhesus macaque	HD (ibotenic acid lesion)	Monkey fetal neostriatal neurons	Striatum	Dense gliosis of degenerating grafts at 8 months post-tp

*Abbreviations: AD, Alzheimer's disease; GDNF, Glial cell line derived neurotrophic factor; HD, Huntington's disease; PD, Parkinson's disease; tp, transplantation; 6-OHDA, 6-hydroxydopamine*.

## Deep brain stimulation and the glial response

In the last 20 years, DBS has been used to treat a range of neurological disorders, most notably in patients with advancing PD and those with essential tremor refractory to medical therapy (Lyons, 2011). DBS involves applying high frequency stimulation (HFS) via implanted electrodes into strategic CNS nuclei which serves to modulate the output of these nuclei with therapeutic benefits in the majority of patients. This benefit persists for many years and has encouraged the adoption of this approach for a whole range of new indications including neuropsychiatric conditions and pain, as well as in earlier stage PD. However, the host CNS glial response to the implantation of this foreign body and the HFS that it delivers has been the subject of only a few reports, especially in the clinical field.

In contrast, there has been a significant amount of experimental work over the years to look at how the CNS reacts to chronically implanted electrodes, although more often in the context of micro-recording than DBS. This is because micro-recording is a well-established method for studying CNS function and DBS has been developed for use primarily in non-human primates and man rather than rodents. Nevertheless, as one would expect, the insertion of a metal electrode into the parenchyma of the brain will induce an astrocytic response (both in terms of the number and degree of astrocytes activated), the magnitude of which may be dependent, in part, on the electrode architecture (Szarowski et al., 2003; Groothuis et al., 2014), and the biomaterial being used (Ereifej et al., 2011). Studies of this type have led some to look at non-metallic electrodes with the hope that this will induce less of a glial response (Ereifej et al., 2011). For example, stainless-steel, as oppose to platinum-iridium or tungsten electrodes, are more prone to electrolytic dissolution which may increase the likelihood of local cell death and a secondary astroglial response (Harnack et al., 2004).

In all cases, the astrocytic and microglial responses—which typically include a change in cell morphology and density of activated cells as well as the release of various pro-inflammatory molecules—are evident soon after implantation (Figure 1.1), persist for years (Jarraya et al., 2003) and seem to occur independently of where the electrode is placed in the brain and the clinical condition for which it is being used (Moss et al., 2004). The extent to which this reactive astroglial and microglial response changes over time is unknown (although see Griffith and Humphrey, 2006), as are the effects that such changes have on the efficacy of current delivered by the stimulating electrode. Nevertheless, a number of experimental studies have shown that these glial reactions could adversely affect electrode impedance and thus efficacy (Spataro et al., 2005; Polikov et al., 2009; Frampton et al., 2010). Indeed, there is even some evidence that chronically implanted electrodes cause sustained local inflammation and neurodegeneration (McConnell et al., 2009). For example, in a study performed in rodents, McConnell et al. demonstrated that heightened levels of chronic inflammation correlated with increased neuronal and dendritic loss, a phenomenon which was more pronounced at 16 weeks than at 8 weeks post-implantation. Remarkably, this local but progressive neurodegeneration was accompanied by axonal pathology, as evidenced by tau phosphorylation, a prominent feature of neurodegenerative conditions. Exactly how this tau pathology arises is unclear but it does suggest that inflammation may be able to induce some forms of proteinopathy.

**Figure 1:**
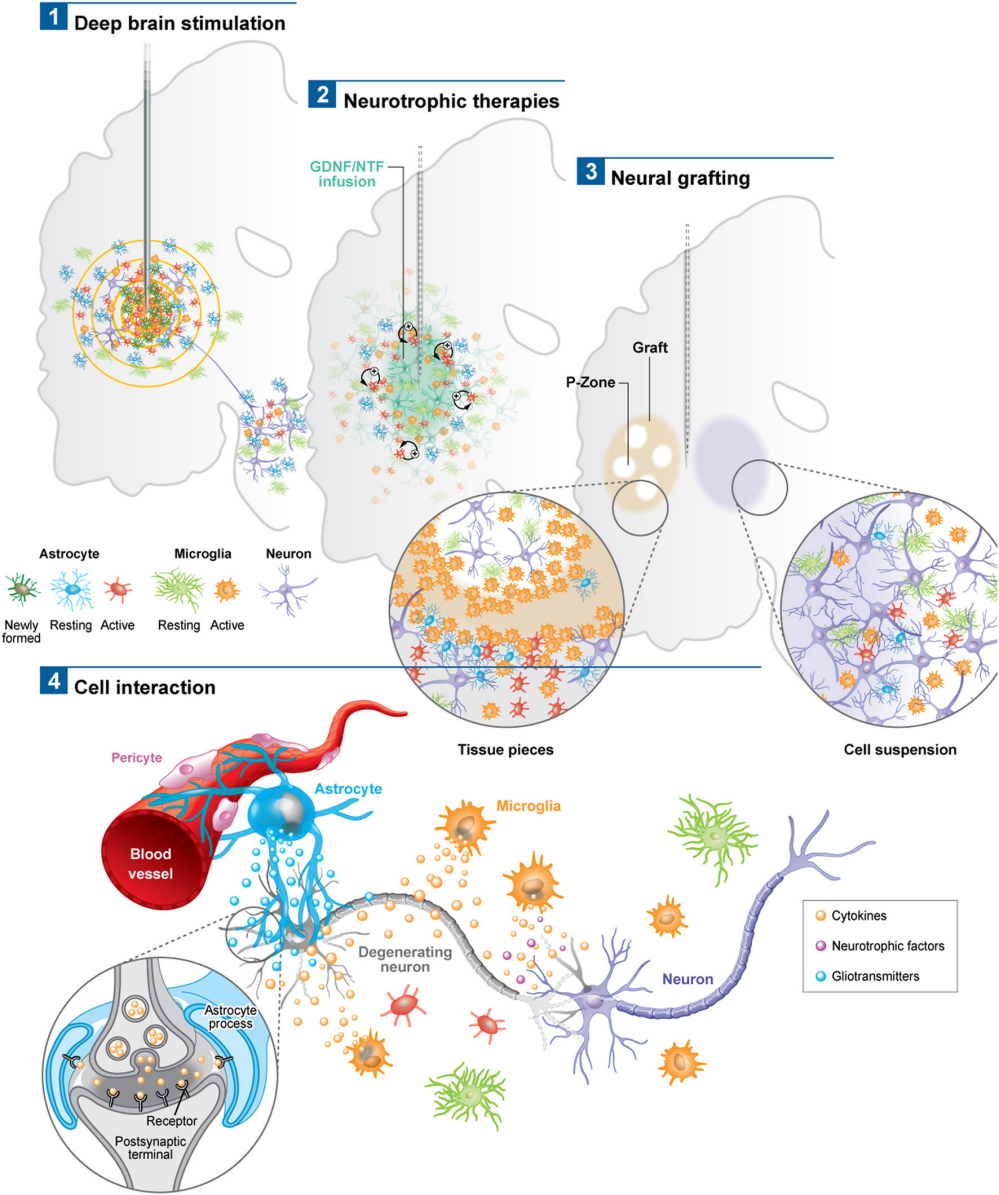
**Schematic depicting the astroglial and microglial responses to a variety of invasive experimental therapeutic approaches being trialed for neurodegenerative disorders (1–3)**. (4) Potential cell interactions that may further influence the outcome of the therapeutic intervention, or, that could be used to potentiate the effects of these therapies. For example the astrocytic response may lead to the release of trophic factors, the modulation of local blow flow as well as the activation of axonal circuits via tripartite synapses. The microglial response may produce both neuroprotective and neurotoxic products, all of which may impact not only on local neurons but also on local vasculature to influence the efficacy of the delivered therapeutic agent. Note: *p*-zone refers to areas of the graft expressing markers for striatal cells.

Additionally, in a recent paper by Vedam-Mai et al. they have interestingly shown in patients that there are variable responses to chronic DBS electrodes, ranging from minimal astrocytosis and microglia activation in the majority of cases to the formation of a dense collagenous band at the electrode tip in at least one case (Vedam-Mai et al., 2012b). In this paper, the authors comment on the possibility of gliotic encapsulation altering electrical thresholds of the stimulating electrode and with this its clinical efficacy—although this study would suggest that this is a rare event. Understanding and predicting such tissue responses to the stimulating and recording electrodes, or even just cannula guidewires, is critical as the magnitude of the foreign body response can lead to electrode failure (Groothuis et al., 2014) and suboptimal clinical outcomes. However, in this study, they did not look to see whether the inflammation induced altered the pathology within the brain, which is of course not straightforward in patients with pre-existing neurodegenerative disorders.

Based on data of this type, some experimental studies have sought to minimize this microglial and inflammatory response, or so-called foreign body response, by using agents such as minocycline and steroids in combination with the stimulating electrode (Rennaker et al., 2007; Zhong and Bellamkonda, 2007) as well as nanoparticle delivery systems (Kim and Martin, 2006; Mercanzini et al., 2010). These different strategies are all predicated on the grounds that suppressing the microglial/inflammatory response to DBS will improve the brain-electrode interface in these chronically implanted micro-electrodes and thus maintain efficacy. Whether this is really a problem in patients with DBS is unknown (see above and Vedam-Mai et al., 2012a,b), but one interesting study by Hirshler et al. (2010) (see also Table 1) in rats showed that merely inserting an electrode into the brain could induce widespread and chronic (i.e., over weeks) neuroinflammation which was correlated with deficits in cognitive function—deficits which are also seen in patients who have had DBS (Witt et al., 2008). No such studies using microglia markers and positron emission tomography (PET) have been performed clinically, although a recent study found that in patients with DBS of the pedunculopontine nucleus, there was an improvement in cognition in association with improvements in cortical activity as measured by fluorodeoxyglucose (FDG)-PET (Stefani et al., 2010). This aspect of DBS, namely its ability to induce widespread inflammation (i.e., not just at the site of implantation of the electrode) across whole neural networks may prove to be much more important in understanding the positive and negative effects on cognition in the large number of conditions for which it is now being used. However, this is clearly complicated by the fact that the stimulation itself will alter the function of neural networks directly, as has been seen in studies looking at cognitive function in patients in receipt of subthalamic DBS (Funkiewiez et al., 2006).

However, more recently, others have argued that the induction of a significant astrocytic response actually enhances any DBS-mediated effects (Fenoy et al., 2014). Fenoy et al. suggest that the inevitable stimulation of astrocytes by DBS can set in motion the release of gliotransmitters (e.g., glutamate, adenosine, and D-serine) that can in turn trigger axonal activation which mediates part of the therapeutic benefits of the stimulation. For example, application of adenosine A1-receptor antagonists abolishes HFS-induced suppression of thalamic neuronal activity. More specifically, adenosine, which results from the conversion of ATP by astrocytes, significantly increases in the electrodes' surroundings, endorsing its role in stimulation-mediated effects on tremor (Bekar et al., 2008). In support of this, the release of glutamate and adenosine both terminate oscillations generated by HFS in thalamic sites (Tawfik et al., 2010).

Astrocytes, which have the capacity to interact with up to 2 million synapses in the human brain, can have a profound effect on synaptic activity as well as other astrocytes across large neural networks (Oberheim et al., 2006). The contact that astrocytes have with blood vessels also uniquely enables them to regulate local blood flow in response to increased neuronal activity, which again may help explain the beneficial effects of DBS (Takano et al., 2006). Using *in vivo* two-photon imaging and photolysis, the authors elegantly demonstrated the association between increases in Ca^2+^ levels in astrocytic end feet with vasodilation, a process which more specifically involves COX-1 metabolites. Finally, DBS can also promote the proliferation of “neurogenic astrocytes” which can differentiate into functional neurons (Vedam-Mai et al., 2012a) (Figure 1). Taken together, this data suggests a prominent role for astrocytes in DBS-mediated effects implying that there may be an optimal balance in the astrocytic response, in terms of positive and negative effects on the stimulated neural pathways, which may in part be responsible for determining the clinical success of this treatment.

In summary, DBS electrodes will induce an astrocytic and microglial response (Figure 1.1) although the extent to which this adversely affects their efficacy in patients is unproven. Indeed, recent data may even suggest the opposite (Moro et al., 2010). However, the data is limited and there is therefore a need to collect systemically more information in this area and moves are afoot to do this (Vedam-Mai et al., 2011), with the expectation that this will enable us to better understand this hitherto poorly described effect of DBS (Vedam-Mai et al., 2011).

## Neurotrophic therapies and the glial response

### Gene therapy

There are a number of gene therapy trials that have been undertaken in PD which seek to either improve the delivery of dopaminergic stimulation to the striatum; restore to normal the abnormal circuitry of the basal ganglia or promote host repair through rescuing dopaminergic neurons and their striatal innervation (reviewed in Berry and Foltynie, 2011). Other gene therapy trials have targeted HD (Bloch et al., 2004) and Alzheimer's disease (AD) (Mandel, 2010). In this last respect, Nerve growth Factor (NGF) transfected fibroblasts were used and one patient died shortly after receiving the therapy for unrelated reasons. At post-mortem, the brain showed surviving cells and NGF expression, and although the glial response to the therapy was not explicitly studied, they did comment there was a minimal inflammatory and glial response, as evidenced by the fact that they found a few granulomatous cells only (Tuszynski et al., 2005).

In the PD studies, again only a few patients have come to post-mortem, so any data as to the host response to these virally delivered agents is very limited and most have concentrated on the extent of tyrosine hydroxylase (TH) fiber sprouting and the volume of distribution of the therapeutic agent, as well as systemic toxicity and local inflammation (e.g., Herzog et al., 2008, 2009; Bartus et al., 2011). However, there are studies showing that these agents may have a primary effect on the astrocytic compartment and that this is the route by which they actually rescue neurons. For example, Hauck et al. (2006) showed that GDNF works indirectly on photoreceptors to rescue them via Mueller glial cells, and so unlike other invasive therapies, part of their efficacy may actually be enhanced through the host glial compartment.

Overall, evidence to date would suggest that the viral delivery of neuroactive substances to the CNS induces very little glial and inflammatory cell responses, including AAV2-Neurturin (Jeff Kordower; Personal Commun.) (Figure 1.2) as well as pre-clinical reports pertaining to this field (Table 2).

### Direct neurotrophic factor infusion

The direct infusion of GDNF into the brain of patients with PD has been the subject of a number of trials. This was initially undertaken using an intraventricular delivery approach that proved ineffective (Nutt et al., 2003), almost certainly because the agent failed to reach the dopaminergic neurons and their projections. This would be consistent with the single post-mortem case from this study showing no evidence of regeneration within the affected nigrostriatal pathway (Kordower et al., 1999). This was then followed by a number of studies in which GDNF was directly infused into the brain parenchyma with variable efficacy—the open label studies showed benefits whilst a double blind placebo controlled study showed no such effects (Gill et al., 2003; Patel et al., 2005; Slevin et al., 2005, 2007; Lang et al., 2006). Although there are various reasons as to why this may have occurred (Barker, 2006), it is of interest to know whether the infusion provoked a glial reaction. This is important not only to see the extent to which chronically implanted catheters delivering neuroactive agents induce a glial response but, given that astrocytes themselves can actually release neurotrophic factors (e.g., Iravani et al., 2012), it is also critical to know whether they may also be capable of enhancing any regenerative response.

In the single post-mortem case looking at intra-parenchymal GDNF delivery in a patient with PD, there was evidence of host TH fiber sprouting around the catheter tip with a low grade astrocytic, microglial and T-cell response (Love et al., 2005). This patient showed a 38% improvement in their contralateral UPDRS (Unified Parkinson's disease rating scale) “off” motor score with a 91% increase in Fluoro-dopa signal in the infused posterior putamen. He died of a myocardial infarction, 3 months after stopping the GDNF therapy that he had been receiving for 43 months. Neuropathologically, there was a slight astrogliosis around the catheter track and tip with a few MHC class II expressing microglia and where this was most intense, there was a reduction in the synaptic protein, synaptophysin. Overall, the glial response to the chronic infusion of GDNF was localized.

## Neural grafting and the glial response

The experimental approach of cell transplantation is one in which the glial response has been more specifically addressed. Two distinct types of cell transplantation have been tested in the clinic—cell suspensions, prepared by mechanically dissociating the cells prior to implantation (Lindvall et al., 1990; Mendez et al., 2002; Freeman and Brundin, 2006) and solid grafts, where donor tissue is transplanted as small pieces (Freeman et al., 1995, 2000; Kordower et al., 1997; Olanow et al., 2003; Freeman and Brundin, 2006; Cicchetti et al., 2009). Each of these strategies is associated with a different pattern and intensity of gliosis. In addition, different cell types may induce different host responses, depending on their source of origin, preparation, and mode of implantation (see Table 3).

In PD, various post-mortem analyses conducted in transplanted patients have shown that solid grafts evoke a more robust and durable immune response in comparison to cell suspensions (Kordower et al., 1997; Olanow et al., 2003; Mendez et al., 2005, 2008; Freeman and Brundin, 2006; Cooper et al., 2009), a finding which is further supported by observations collected in grafted animal models of the disease (Leigh et al., 1994; see also Table 3). Few microglial cells are observed around the needle track with only a mild response around the graft deposits themselves. Similarly, the astrocytic response, which accompanies cell suspension grafts, is predominantly confined to the borders of the graft, with the core of the transplant largely devoid of this cell type (Figure 1.3).

Histological analyses in HD patients transplanted with solid embryonic neural grafts show microglial activation, particularly around the transplant and within the grafted areas expressing striatal markers (referred to as p-zones). Importantly, this significant microglial cuffing is still present 12 years following transplantation (Cicchetti et al., 2009). As observed in cell suspension grafts, solid piece transplants induce little astrogliosis although a number of cells of a reactive phenotype are found around the grafted tissue (Cisbani et al., 2013) (Figure 1.3). This pattern of astroglial activation and distribution may have important implications for the survival of the grafts long-term, as astrocytes play not only a critical role in growth factor release but also in glutamate buffering, and thus excitotoxicity (Cicchetti et al., 2011).

Comparing the post-mortem analysis of HD and PD allografted tissue, irrespective of the type of graft implanted, there appears to be a distinctly different glial reaction. In HD, there seems to be a much more aggressive glial (both in terms of the number of activated cells and their phenotype) response whilst in PD, the evidence would suggest that the microglial response is much less obvious. Therefore, transplantation in different diseases may produce different glial responses which in turn may influence the long term survival, integration and outcome of the grafted issue (Cicchetti et al., 2011, 2014).

Furthermore, we have recently shown the presence of mutant huntingtin protein aggregates within genetically unrelated grafted tissue in HD patients. This observation, which is similar to that reported earlier with the discovery of lewy bodies in grafted fetal ventral mesencephalon tissue in PD patients, could also result, at least in part, from oxidative stress and the host inflammatory response induced by the graft (Li et al., 2008; Cicchetti et al., 2014).

Taken together, it is possible that the host response to solid grafts in the absence of immunosuppression (which was restricted to the first 6 months following transplantation in those HD post-mortem cases described above) generates enhanced antigenic stimulation and a stronger immunological/inflammatory response than with suspension grafts (Kordower et al., 1997; Olanow et al., 2003; Mendez et al., 2005, 2008; Freeman and Brundin, 2006; Redmond et al., 2008). Indeed, the type and duration of immunosuppressive treatment is likely to contribute to some of the discrepancies in the immune/glial responses observed using these different transplants. Nevertheless, the accumulating evidence of an attenuated glial response following transplantation of cell suspension grafts favors their use in future clinical cell transplant programs in patients with neurodegenerative disorders (Freeman and Brundin, 2006).

## Conclusion

There are now a number of new therapies emerging for the treatment of chronic neurodegenerative disorders of the CNS which are invasive but seek to restore to normal the dysfunctional circuits that underlie these conditions. These approaches have generally used a targeted intracerebral delivery of a neuroactive substance or therapeutic device. To date, these therapies have produced mixed results and many explanations to account for this have been put forward including patient selection, disease stage and subtype, mode of delivery, dose given, trial design, and the nature and timing for the primary end-points. However, another area that requires further investigation is the glial and inflammatory response to the novel therapeutic agent as this may impact on its efficacy. To date there are very few studies looking at this and in this review we have sought to highlight this with reference to the limited literature on the microglial and astrocytic reactions to these therapies. At the present time, such responses seem likely to be less of an issue with growth factor infusions and gene therapies—although this may simply reflect the limited post-mortem analyses on this to date—but may be highly relevant for the short and long term viability of neural transplants as well as DBS efficacy, and to a greater magnitude than originally thought. The only way such information can meaningfully be obtained is through the detailed analyses of the post-mortem cases that can be made available by such trials and which will allow us to understand which cells are affected and to what extent. Indeed, one of the critical questions will be the extent to which each cell type influences the behavior of another, as it is now clear that there is a dialog between the glial cells, neurons, and vasculature (Figure 1.4). An improved understanding of these interactions may ultimately impact on our ability to better treat patients using these novel approaches as well as modifications as to how they are can be optimally delivered.

### Conflict of interest statement

The authors declare that the research was conducted in the absence of any commercial or financial relationships that could be construed as a potential conflict of interest.
